# Supplementary figures and images for: Limited hyperoxia-induced proliferative retinopathy: A model of persistent retinal vascular dysfunction, preretinal fibrosis and hyaloidal vascular reprogramming for retinal rescue
Source: PLoS One. 2022 Apr 27;17(4):e0267576. doi: 10.1371/journal.pone.0267576 (PMC9045626; doi:10.1371/journal.pone.0267576)

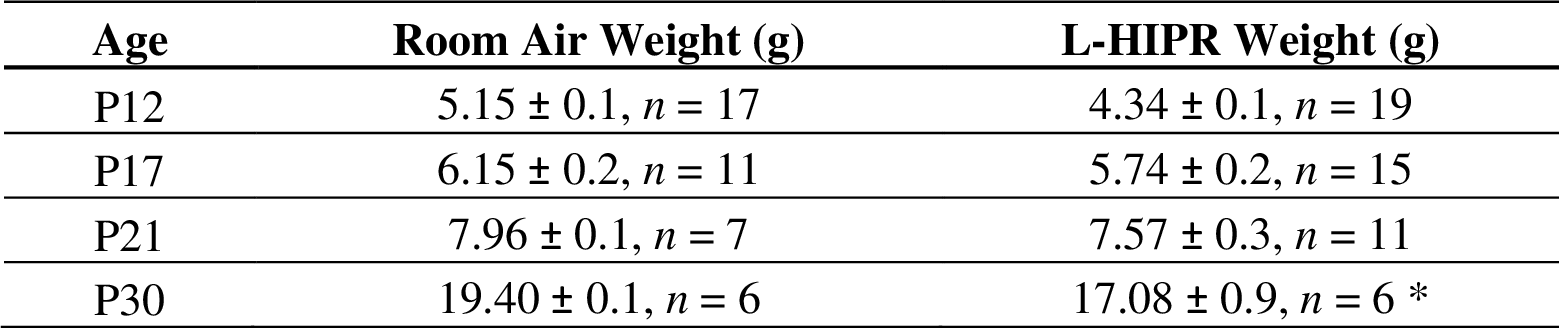

Supplement: S1 Table — Significantly lower weight gain was detected at P30 in L-HIPR group, but average litter weights were similar to room air controls before these neonates were weaned. Values presented as mean weight (g) ± S.E.M., *: p < 0.05, one-way ANOVA with Bonferroni post hoc analysis. (TIF) [file pone.0267576.s002.tif]

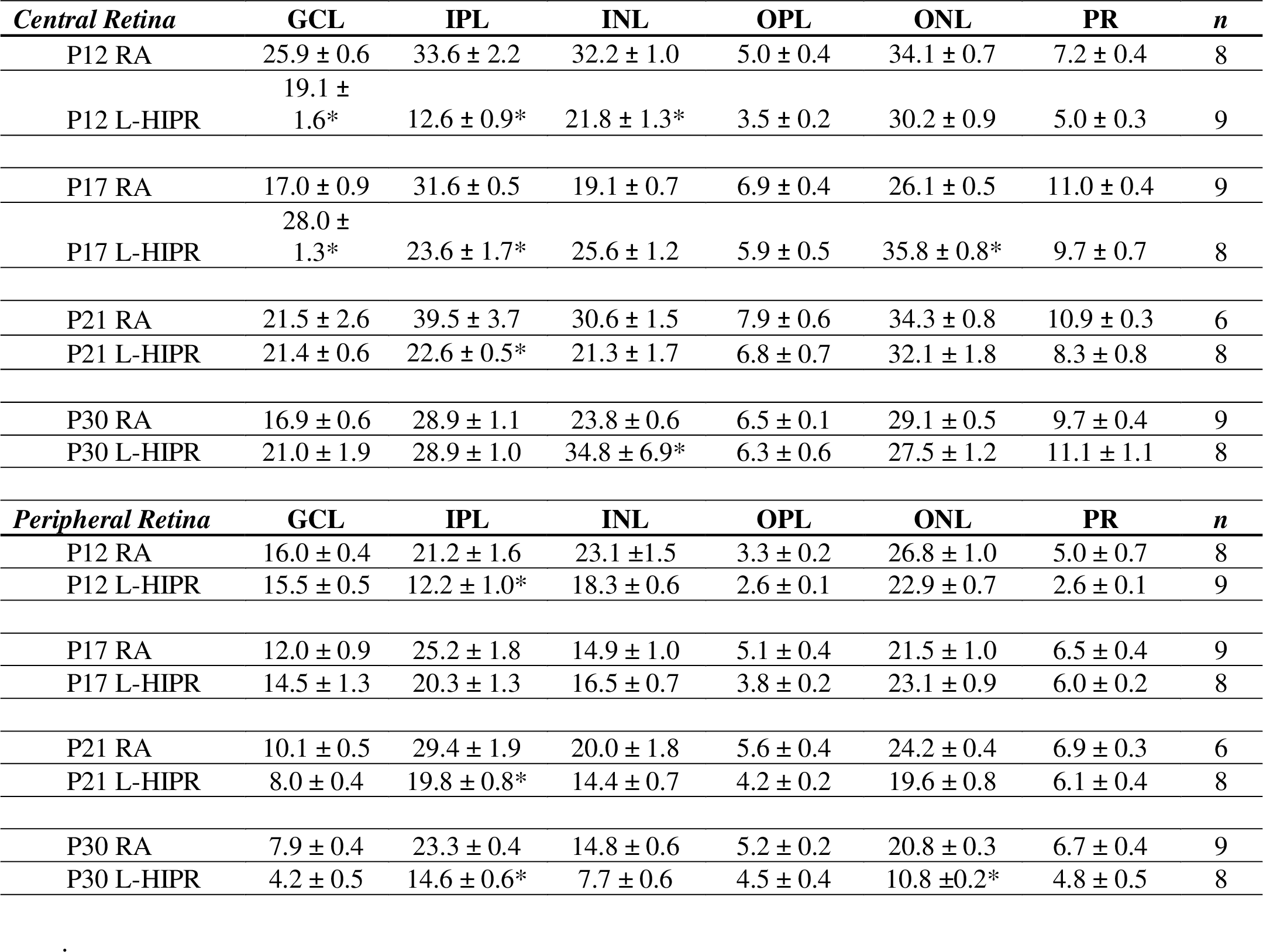

Supplement: S2 Table — We compared the room air and L-HIPR retinal layers at P12, P17, P21, and P30 by measuring central (near the optic nerve) and peripheral regions. (TIF) [file pone.0267576.s003.tif]

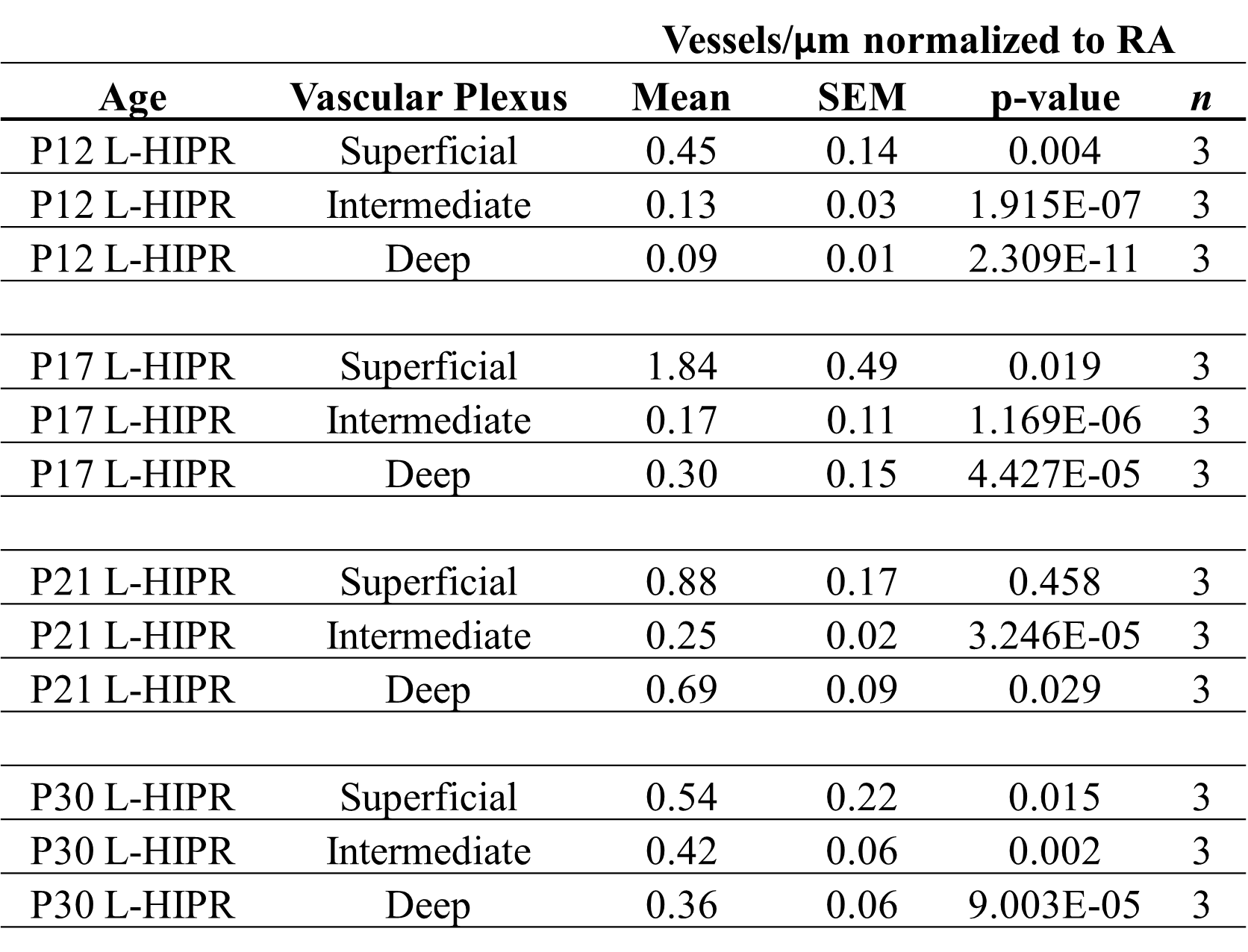

Supplement: S3 Table — Quantification of blood vessel density within the 3 vascular plexuses in L-HIPR normalized to age matched room air control. (TIF) [file pone.0267576.s004.tif]

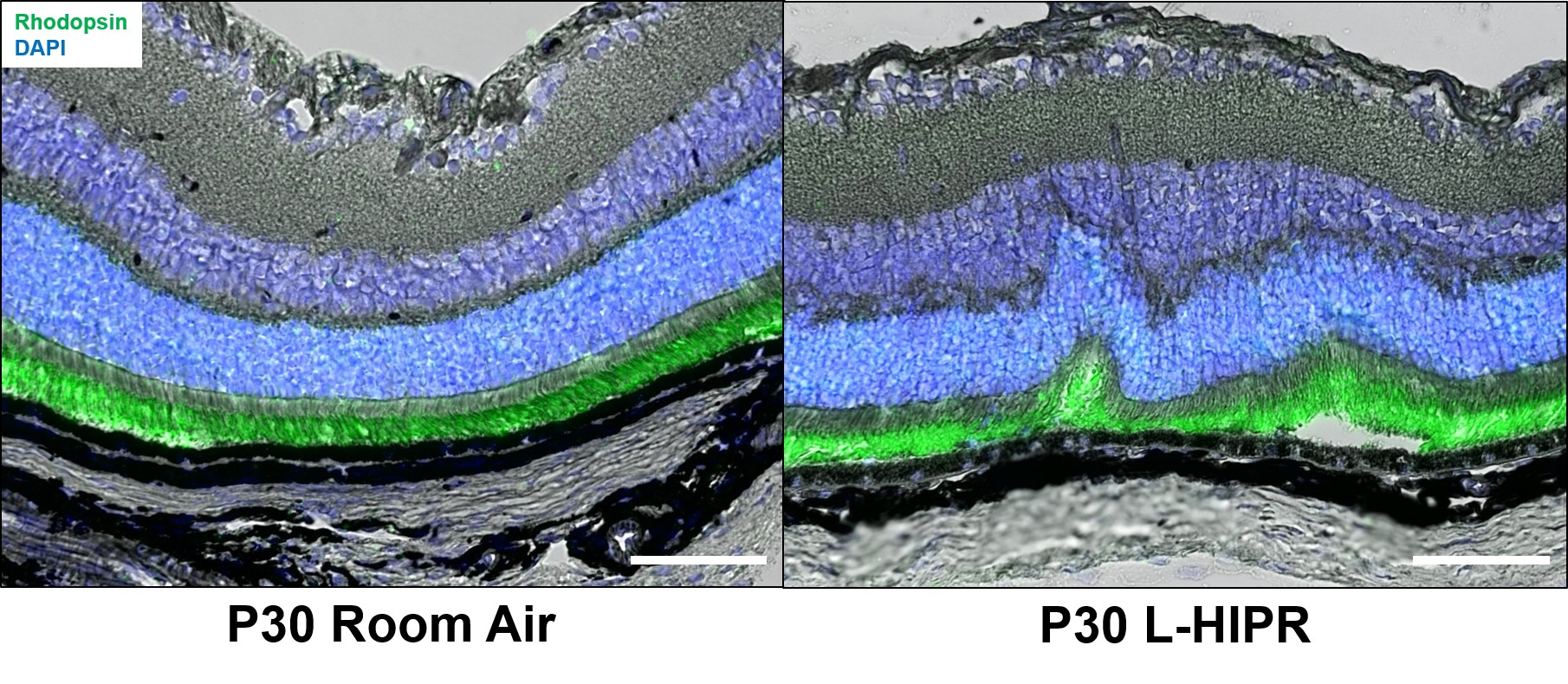

Supplement: S1 Fig — Immunolabeled paraffin cross-sections labeled with anti-Rhodopsin (green) and nuclei with DAPI (blue) overlayed with differential interference contrast microscopy. Scale bars equal 50 μm. (TIF) [file pone.0267576.s005.tif]

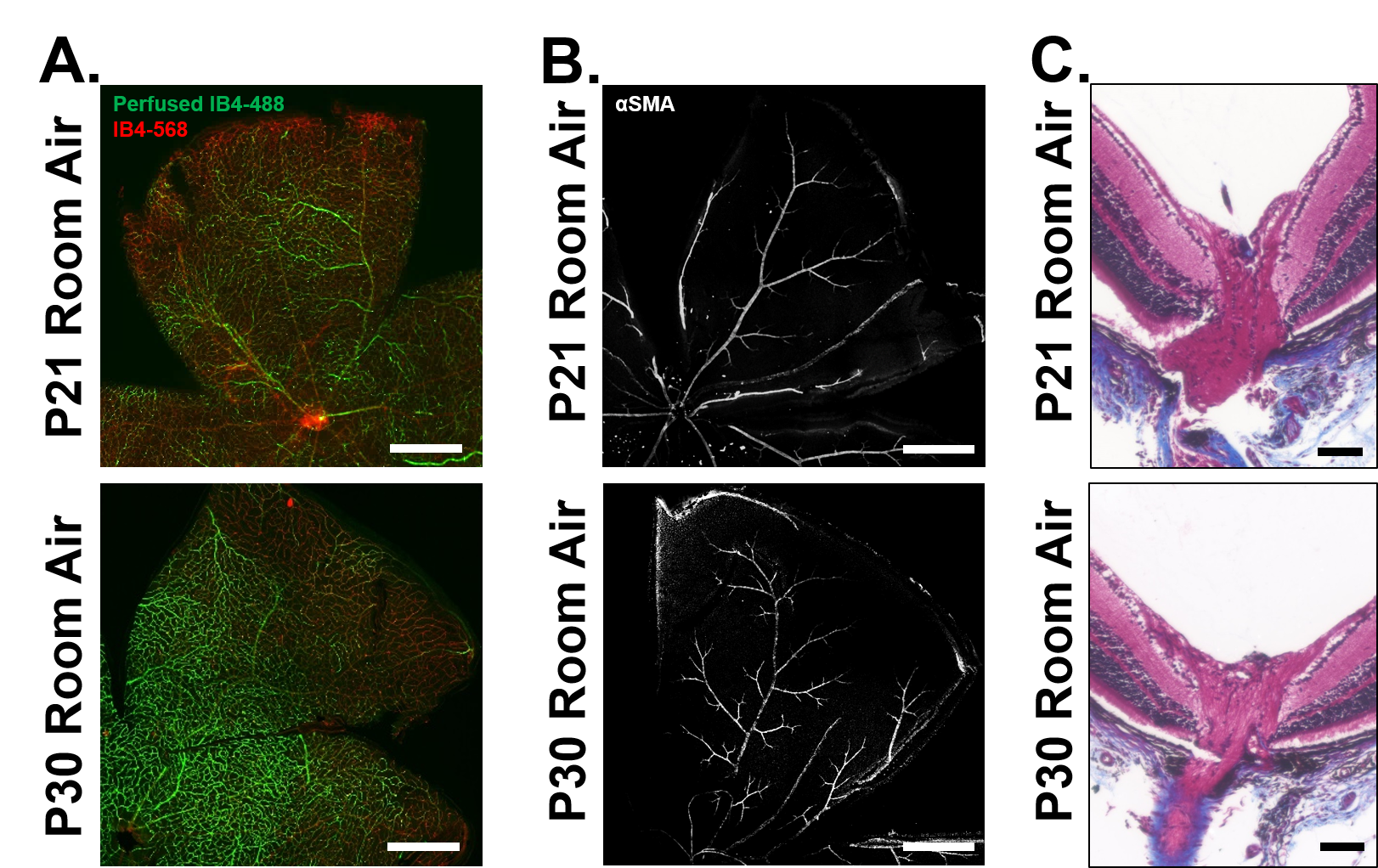

Supplement: S2 Fig — (A) IB4-488 cardiac perfusion (B) α-SMA and (C) Masson Trichrome staining. Scale bars equal 300 μm in (A) and (B), 50 μm in (C). (TIF) [file pone.0267576.s006.tif]
